# Stable Metabolic Control but Increased Demand for Professional Support in Children with Type 1 Diabetes in the Past Ten Years in Bern/Switzerland: A Quality Control Study

**DOI:** 10.1155/2022/3170558

**Published:** 2022-08-17

**Authors:** Michelle J. Dennig, Grit Sommer, Tanja Zingg, Christa E. Flück, Claudia Boettcher

**Affiliations:** ^1^Department of Paediatric Endocrinology, Diabetology & Metabolism, University Children's Hospital, Inselspital, Bern, Switzerland; ^2^Department of BioMedical Research, Bern University Hospital and University of Bern, Bern, Switzerland

## Abstract

**Introduction:**

Lower HbA1c targets and increasingly complex diabetes management with substantially increasing costs dominate today's type 1 diabetes therapy in children and adolescents.

**Objective:**

To evaluate metabolic control in children and adolescents with type 1 diabetes and assess associated factors, evaluate determinants for frequency of healthcare contacts, and compare actual with historical data.

**Method:**

This cross-sectional observational study collected data on 178 children and adolescents with type 1 diabetes treated at the University Children's Hospital in Bern.

**Results:**

Mean HbA1c was 7.9% (63 mmol/mol), 33.1% (59/178) of children reached the target of HbA1c < 7.5% (<59 mmol/mol), and 18.0% (32/178) had an HbA1c value < 7.0% (<53 mmol/mol). Compared to historical data, stable HbA1c levels appeared with a doubled proportion of individuals using insulin pumps. Metabolic control was worse with a longer duration of diabetes and younger age at diagnosis but better when parents came from a Western European country. Age at the consultation, use of diabetes technology and native language influenced the number of healthcare contacts. Younger patients, patients using CSII, and patients without an official Swiss language as mother tongue had more consultations with a healthcare professional than older and native language individuals.

**Conclusion:**

The metabolic targets in childhood and adolescent type 1 diabetes are still unmet despite a shift towards more technology. Our study documents a higher demand for support and supervision in specific patient groups. An investment to increase healthcare contacts could help combat the increase in total diabetes cost and significantly improve metabolic control.

## 1. Introduction

The declared goal of diabetes therapy—formalised in guidelines—is to achieve the best metabolic control possible without risking acute and chronic disease complications. As a measure for glycaemic control, HbA1c plays a significant role in monitoring diabetes treatment and management. The guidelines' recommendations for HbA1c targets in children and adolescents differ worldwide and have changed in the last decade, with target HbA1c levels varying between <6.5 and 7.5% (<48-59 mmol/mol) [1–3]. Before 2018, target levels were <7.5% (<59 mmol/l) recommended by the International Society for Pediatric and Adolescent Diabetes (ISPAD) [3] and the American Diabetes Association (ADA) [1]. Today, both the ISPAD and the ADA recommend HbA1c values < 7.0% (<53 mmol/mol) [4]. The most stringent targets for children and adolescents are recommended by Sweden and the UK, with a target of <6.5% (<48 mmol/mol) [2].

Over the last decades, various new therapeutic options, especially technical devices, for children and adolescents with type 1 diabetes emerged. These new therapeutic options promised better metabolic control and better quality of life as discussed by Müller-Godeffroy et al. [5]. However, these resulted in increased costs of type 1 diabetes [6–8] due to the cost of the technical devices and of the additional time needed, e.g., for a higher number of training sessions with patients.

This study is aimed at describing adherence to HbA1c targets, metabolic control, and the involved resources of medical personnel and investigating their influencing factors in children and adolescents with type 1 diabetes. We also aimed to compare metabolic control and treatment modalities with data from 10 years before.

## 2. Methods

### 2.1. Study Design and Data Source

Between 09/2017 and 03/2018, we conducted a single centre cross-sectional observational study within our outpatient centre for paediatric patients with diabetes in Bern as part of our treatment centre's quality control. Each patient's data was recorded only once during the study period. The survey comprised several steps: step one consisted of collecting detailed information on diabetes diagnosis, duration, treatment, metabolic control, and demographic data of all patients during the scope of routine consultations. Step two included registering the number of contacts with physicians and nursing staff within the previous 12 months through our electronic patient management systems. In step three, we compared the 2017/18 data with “historical” Bernese centre data [9] from 2008.

### 2.2. Study Population

Individuals were eligible if they had a diagnosis of type 1 diabetes, were aged <18 years at the time of the study, and were treated at the University Children's Hospital Bern's outpatient centre for paediatric patients with diabetes. Patients without available HbA1c and/or within the first three months of manifestation of type 1 diabetes were excluded. In total, we were able to include data of 178 individuals in the study. The number of healthcare contacts with physicians and nurses was evaluated in individuals with a duration of diabetes ≥ 1 year (*n* = 151). Patients and/or their parents gave informed consent. The Ethics Commission Bern approved our quality control study.

### 2.3. Variables

We collected demographic data, diabetes-related data, and treatment-related data during patients' routine consultations in our paediatric endocrinology unit. The data included current age at consultation, sex, height, weight, language, parent origin, age at the manifestation of diabetes, duration of diabetes, treatment modality, and use of continuous glucose monitoring (CGM). We calculated body mass index (BMI) standard deviation scores (SDS) from references of the Swiss normal population [10]. We coded language as speaking a Swiss national language (German, French, or Italian) or speaking another language. Treatment modalities were conventional therapy (CT), multiple daily injections (MDI), or continuous subcutaneous insulin infusion (CSII). In CT, patients injected insulin twice/thrice daily and followed an individual diet plan with fixed carbohydrates. We coded patients with either real-time or intermittently scanned glucose monitoring as using continuous glucose monitoring (yes/no). We coded age < 5, 5 to <10, 10 to 15, and 15 to <18 years. BMI was categorized according to SD < 2, -2 to -1, -1 to <1, 1 to <2, and >=2. We coded parental origin according to the UN continental regions; when parents were of different origin, we coded according to the parent of non-Western European origin. We subdivided the category European into Western European and other as the majority of our patients were of Western European origin (*n* = 140, 78.7%). We analysed HbA1c values categorized into <7% (53 mmol/mol) and <7.5% (59 mmol/mol). As a measure of time expended for each patient, we recorded the number of healthcare contacts separately for physicians and nurses. Using our medical electronic information systems, we recorded the number of contacts for the 12 months prior to the patients' first consultation. The number of full time equivalents for physicians and nurses (2008 and 2017/18) was recorded.

### 2.4. Statistical Analysis

Continuous variables were described as mean values and standard deviation (SD) and categorical variables as numbers and frequencies in percent. We also expressed continuous variables as median with 25th and 75th percentiles to facilitate comparisons with previous studies. We used a two-sample *t*-test to compare mean HbA1c values between 2008 and 2017/18. We used linear regression analyses to assess the association between clinical characteristics and HbA1c or healthcare contact per year, respectively. All regression models included age and sex as confounding variables. Results from linear regression are indicated as *β*-coefficients and 95% confidence intervals. *P* value < 0.05 was considered statistically significant. We used the statistical software Stata (Version 15, Stata Corporation, Austin, TX) for all analyses.

## 3. Results

### 3.1. Description of the Study Cohort

In total, 178 patients with type 1 diabetes participated in our study. Of those, 92/178 were boys (51.7%), and 86/178 were girls (48.3%) (Table 1). Patients had a mean age (SD) at the consultation of 11.7 (3.6) years and a mean duration of type 1 diabetes of 4.7 (3.6) years. Most patients (62.9%, 112/178) received multiple daily injections (MDI), 26.4% (47/178) received subcutaneous insulin infusion (CSII), and 10.7% (19/178) received conventional therapy (CT). Thirty-nine and three-tenths percent (70/178) of patients used CGM in their diabetes management. In total, CSII (24/47, 51.0%) and CGM (32/70, 45.7%) use was most common in children aged 10 to <15 years, followed by children ages 5 to 10 years for CSII (10/47, 21.3%) and CGM (18/70, 25.7%). However, the percentage in relation to number per age group showed that use of CSII and CGM was highest in children < 5 years with 44.4% for CSII (4/9) and 66.7% for CGM (6/9), followed by CSII in children aged 10 to <15 years (24/90, 26.7%) and CGM in children ages 5 to <10 years (18/42, 42.9%).

### 3.2. Metabolic Control and Healthcare Contacts

Mean HbA1c was 7.9% (63 mmol/mol) with 18.0% (32/178) of patients reaching the ISPAD target of HbA1c < 7.0% (<53 mmol/mol) and 33.1% (59/178) of patients reaching the ADA target from 2018 of HbA1c < 7.5% (<59 mmol/mol). Children with a younger age at type 1 diabetes manifestation and with a longer diabetes duration had higher HbA1c values than children with older age at manifestation and with a more recent diagnosis, respectively (all *P* < 0.05) (Table 2). Children with parents of Western European origin had better metabolic control than parents with other origin. For example, children with parents of non-Western European origin had on average HbA1c values that were 0.43% (95% CI 0.05-0.80) higher than those of children with Western-European parents (reference group). We found no influence of age at consultation, language, use of CGM, and treatment modality on HbA1c values.

Children of younger age at the consultation, who were not native speakers of a Swiss national language, who had parents with other than Western European origin, and who used CSII had more healthcare contacts per year than older children at the consultation, children who were native speakers of a Swiss national language, who had parents of Western European origin, and who used CT or MDI (all *P* < 0.05) (Table 2). For example, children using CSII had on average 2.6 more healthcare contacts than children using MDI (reference group) in a 12 month period.

**Figure 1:**
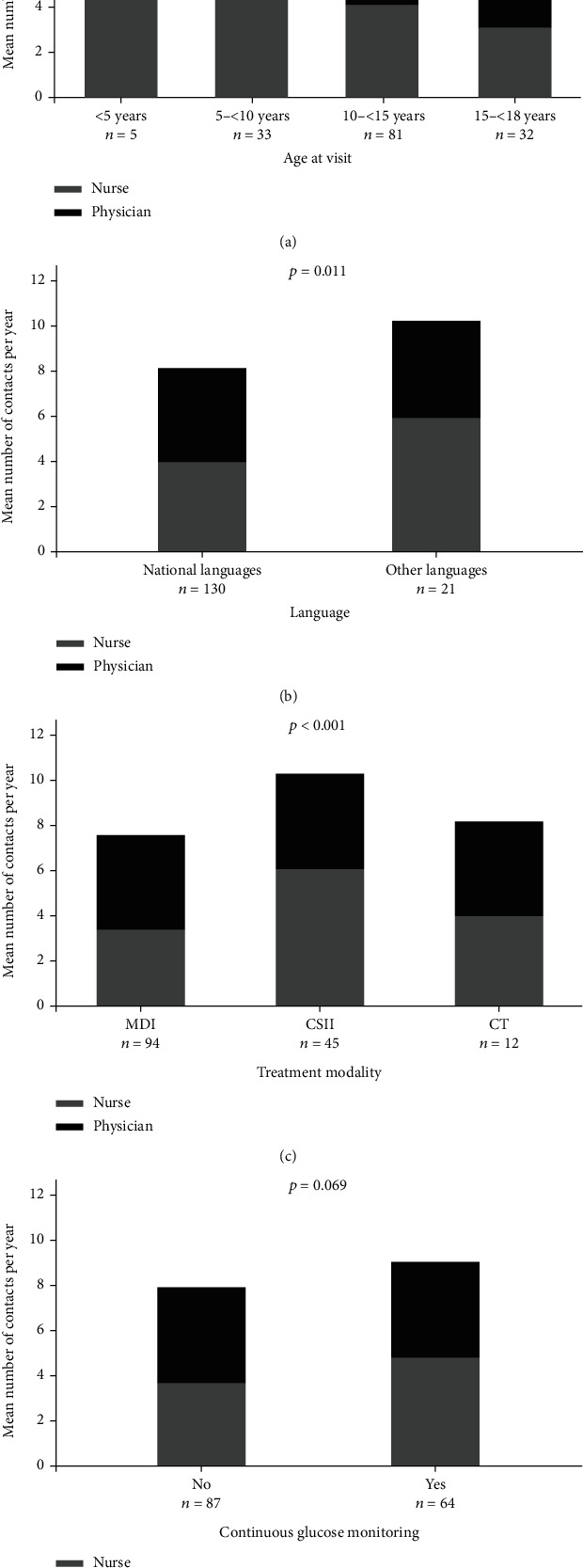
(a–d) Number of healthcare contacts in relation to age at (a) consultation, (b) native language, (c) treatment modality, and (d) use of continuous glucose monitoring. Global *P* values derived from linear regression. Number of healthcare contacts refers only to patients with a type 1 diabetes duration of >1 year (*n* = 151).

The number of healthcare contacts did not differ by sex, duration of diabetes, age at diabetes manifestation, or CGM use (Figure 1).

### 3.3. Comparison between 2017/2018 and 2008

Mean HbA1c values did not differ between 2008 and 2017/18 (*P* = 0.451) (Table 3). Frequencies of CGM and CSII use increased between 2008 and 2017/18.

In both 2008 and 2017/18, three and a half full-time equivalent of physicians worked in our unit, but the full-time equivalent of nurses increased and was threefold higher in 2017/18 than in 2008. Since 2008, the number of consultations with physicians has increased by 21.6%, from 552 consultations per year in 2008 to 671 consultations per year in 2018, while patient load increased by ~18%.

## 4. Discussion

Our study showed that only a third of the individuals with type 1 diabetes participating in our study reached the current 2020 metabolic target set by the ADA. These results are comparable with other countries, where only a minority of patients reach the set metabolic targets. A study comparing metabolic control across eight high-income countries (2013-14) showed a mean HbA1c lying between 7.6% (60 mmol/mol) in Sweden and 8.8% (73 mmol/mol) in Wales [11]; the data was collected using population-based national registries or audits (except for the US), covering >80% of children with type 1 diabetes. Mean HbA1c in five of eight countries in the study was higher than the mean HbA1c reached in our centre. Metabolic control measured as mean HbA1c in Germany, Austria, and Luxembourg (DPV registry) was 7.7%-7.8% (61-62 mmol/mol). Another study involving US Type 1 Diabetes Exchange registry showed that between 2016 and 2018 17% of patients (<18 years) reached the ADA target < 7.5% (<59 mmol/mol) with a mean HbA1c of 8.4% (68 mmol/mol) [12]. Gerhardsson et al. [13] showed in their study using the SWEET registry that 34% patients were able to meet the more stringent target HbA1c of <7% (<53 mmol/l). In the time period 2016-2018, mean HbA1c across patients < 25 years was 7.5% (59 mmol/mol). We can see room for improvement worldwide, as not only our centre is struggling to reach the set metabolic targets.

When considering which patient groups had worse HbA1c, we saw that younger children at diabetes manifestation had worse metabolic control than older counterparts. Patients with parents of non-Western European origin and those with longer diabetes duration had higher HbA1c levels than patients with Western European origin and those with shorter diabetes duration. Younger patients also needed more healthcare contacts per annum than older patients. The increased need for support in younger patients is not surprising: caregivers of young children face many challenges in achieving the targeted metabolic control without severe hypoglycaemia. The burden of responsibility for diabetes management is exceptionally high in the caregivers of young children, as discussed by Burckhardt et al. [14]. Glycaemic control in the young age group is influenced by many factors in daily life, including responsibility of diabetes management, for instance when management is carried out through day care or school staff. To improve metabolic control in this vulnerable age group, we consider that an increased demand for support exists and more may need to be done in educating all adults involved in the diabetes management of young children.

In individuals with longer diabetes duration and thus increasing age, the transfer of responsibility for diabetes management creates challenges between (pubertal) patients and their parents which results in worsening of HbA1c levels [15, 16]. In our outpatient care, we must try to guide this shift in responsibility between parent and patient without deterioration of metabolic control. In our study, we also saw increasing HbA1c levels with increasing age, but statistical tests found no difference. Perhaps due to low patient numbers in the age groups, we may have missed these differences.

A migratory background is a predictor of poor metabolic control as previously shown [17–19]. Although individuals with a non-Western European origin had more healthcare contacts in our study than individuals with Western European origin, HbA1c was still worse. To attain better metabolic control, high healthcare contact frequency and increased supervision are necessary, together with interpersonal skills. So far, we have not been able to find the right strategy to improve metabolic control in patients with a migratory background.

We saw that the number of healthcare contacts increased with the use of CSII, demonstrating the complexity of using diabetes technology. Assistance by healthcare professionals is necessary to ensure the best utilization of diabetes technology devices. The increase in diabetes technology has been seen in many countries and is especially noteworthy in younger children. The T1D Exchange showed CGM use rising from 7% (2010-2012) to 30% (2016-2018), and insulin pump use increased from 57% to 63%. Both CGM and CSII use increased the most in children < 6 years of age [12]; the Prospective Diabetes Follow-up Registry (DPV) showed a similar effect between 2011 and 2016. Continuous glucose monitoring use increased from 4% to 19% in total for all age groups, with the most significant increase in children < 6 years of age. The use of CSII also increased from 43% to 56% [20]. As the raw data from the study from 2008 no longer existed, we could not comment on which age group had most increase of CSII and CGM use.

Diabetes technology is advantageous in paediatric diabetes care. CSII and CGM use both have been shown to improve metabolic control in multiple studies and reduce severe hypoglycaemia [13, 21–23]. We were not able to show an improved metabolic control using CSII and CGM in our centre; we speculate that this is due to our small patient number. Additionally, the use of CSII and CGM improves the quality of life for patients and their families [24].

Nonnative speakers of a Swiss language needed more healthcare contacts per year than patients speaking a native language, but fortunately, both groups had similar metabolic control. This fact highlights that increased support is necessary for these patients, probably due to the language barrier, to ensure a comparable metabolic control. We plan to continue to accommodate this need in patients whose native language is not a Swiss native language.

In our study, we could not show that metabolic control was positively influenced by the use of CGM or CSII—probably due to small patient numbers. However, we saw an increase in the need for healthcare contacts in these patient groups. In parallel, we saw an increase in the full-time equivalent of nursing staff since 2008 and an increase in healthcare contacts per year with physicians. While ISPAD and the German Diabetes Association (DDG) do not specify the number of healthcare contacts per year, both recommend assessment of metabolic control (HbA1c) at least every three months [3, 25].

Our tertiary centre study shows that between 2008 and 2018, we had a stagnating HbA1c—despite putting a lot of time and effort into improving our patient care. This result highlights the importance of regular quality control of diabetes management (e.g., through assessments of mean metabolic control and beyond) in a diabetes centre. To improve mean HbA1c in our patients, we have implemented several changes. Since evaluating these results, our clinic has joined the DPV registry (http://www.d-p-v.eu/), which provides biannual monitoring on diabetes care and metabolic control of our patients. We counsel patients on methods to correct hyperglycaemia to a lower target and encourage them to consider using CGM and CSII in their diabetes management.

Our study has several limitations: it was a single-centre study with a relatively small number of participants, and we may have missed minor effects because variability was high. Data collection was retrospective, and the recorded parameters were limited and could not be extended. Ideally, we would have collected more data and included more information on the socioeconomic background, necessity of a translator in patients speaking a nonnative language, and in the case of individuals using isCGM, number of scans per day. Although patients characteristics' were comparable between the two time periods, we could not do further statistical tests comparing our study populations between 2008 and 2017/2018, as the raw data from 2008 no longer existed. Even though the data is older, it is valuable for comparison.

In conclusion, we could show that certain patient groups have higher demands for support and supervision. We saw that particularly younger patients and patients using CSII required more healthcare contacts. These patients, patients with non-Western European origin and patients speaking a nonnative language, need more support in our outpatient clinic. Our attending physicians and nurses plan to accommodate this need in future by scheduling additional consultations to best support our patients speaking a nonnative language and caregivers of younger children. We also encourage use of CSII and CGM in diabetes management. Having joined the DPV registry, we now receive regular feedback on mean metabolic control in our centre. Our aim must be to improve metabolic control through the best possible outpatient care. Through this, we are striving to improve long-term patient health and quality of life. Further research should be done to ascertain patient characteristics with increased needs when considering the future development and expansion of outpatient patient care in type 1 diabetes.

## Figures and Tables

**Table 1:** Characteristics of patients with type 1 diabetes mellitus treated in 2018 at the Children's Hospital in Bern.

	All patients		Boys		Girls	
	*N* = 178		*N* = 92		*N* = 86	
	*n*	%	*n*	%	*n*	%
Age at first consultation (years)						
<5	9	5.1	5	5.4	4	4.7
5 to <10	42	23.6	24	26.1	18	20.9
10 to <15	90	50.6	46	50.0	44	51.2
15 to <18	37	20.8	17	18.5	20	23.3
Language						
Official Swiss language	153	86.0	79	85.9	74	86.0
Other language	25	14.0	13	14.1	12	14.0
Continent of parental origin^†^						
Europe	159	89.3	81	88.0	78	90.7
Of these Western Europe	140	78.7	9	9.8	7	8.1
Of these other Europe	19	10.7	68	73.9	61	70.9
Asia	11	6.2	7	7.6	4	4.7
Africa	5	2.8	3	3.3	2	2.3
North America	3	1.7	1	1.1	2	2.3
Body mass index (SDS)						
<-2	3	1.7	2	2.2	1	1.2
-2 to <-1	16	9.0	9	9.8	7	8.1
-1 to <1	129	72.5	68	73.9	61	70.9
1 to <2	26	14.6	13	14.1	13	15.1
>=2	4	2.2	0	0.0	4	4.7
Age at T1DM manifestation (years)						
<5	56	31.5	25	27.2	31	36.0
5 to <10	80	44.9	50	54.3	30	34.9
10 to <16	42	23.6	17	18.5	25	29.1
Duration of T1DM (years)						
<2	50	28.1	24	26.1	26	30.2
>2	128	71.9	68	73.9	60	69.8
Modality of insulin therapy						
MDI	112	62.9	56	60.9	56	65.1
CSII	47	26.4	26	28.3	21	24.4
CT	19	10.7	10	10.9	9	10.5
Continuous glucose monitoring						
No	108	60.7	56	60.9	52	60.5
Yes	70	39.3	36	39.1	34	39.5
HbA1c (%)						
<7.0 (<53 mmol/mol)	32	18.0	18	19.6	14	16.3
<7.5 (<59 mmol/mol)	59	33.1	32	34.8	27	39.7
	Mean	SD	Mean	SD	Mean	SD
Age at first consultation (years)	11.7	3.6	11.5	3.7	12.0	3.6
Body mass index (SDS)	0.2	0.9	0.1	0.9	0.3	1.0
Age at T1DM manifestation (years)	7.0	3.7	7.0	3.7	7.0	3.8
Duration of T1DM (years)	4.7	3.6	4.4	3.2	5.0	4.0
HbA1c (%)	7.9	1.0	7.9	1.1	7.9	1.0
Number of healthcare contacts^‡^						
With a physician	4.2	0.9	4.3	0.8	4.1	0.9
With a nurse	4.2	3.2	4.2	2.9	4.3	3.6
With a physician and/or nurse	8.4	3.5	8.5	3.2	8.3	3.9

Abbreviations: CSII: continuous subcutaneous insulin infusion; CT: conventional therapy; MDI: multiple daily injections; SDS: standard deviation score; T1DM: type 1 diabetes mellitus. ^†^Parents were from the following regions: Europe (Western, *n* = 140, *n* = 131 of those from Switzerland; Northern, *n* = 1; Southern, *n* = 16; Eastern, *n* = 2), Asia (Western, *n* = 6; Southern, *n* = 4; Eastern, *n* = 1), Africa (Western, *n* = 1; Northern, *n* = 2; Eastern, *n* = 2), and North America (Northern, *n* = 2; Carribean, *n* = 1) according to UN classification. ^‡^Number of healthcare contacts refer only to patients with a T1DM duration of >1 year (all patients, *N* = 152; boys, *N* = 79; girls, *N* = 73). Number of contacts refers to contacts within the previous 12 months.

**Table 2:** Influence of sociodemographic, medical, and treatment-related factors on HbA1c concentrations and on number of healthcare contacts in patients with type 1 diabetes mellitus, results from linear regression adjusted for age at consultation and sex. Reference groups for comparison within categories are labelled with (ref).

	HbA1c (%)				Number of healthcare contacts^†^			
	Coef	95% CI		*P* value	Coef	95% CI		*P* value
Sex				0.997				0.833
Boys (ref)	0				0			
Girls	0.00	-0.31	0.31		0.12	-1.00	1.24	
Age at consultation (years)				0.064				0.013
<5	0.03	-0.72	0.78		2.14	-1.13	5.41	
5 to <10 (ref)	0				0			
10 to <15	0.50	0.11	0.88		-1.26	-2.67	0.15	
15 to <18	0.27	-0.19	0.73		-2.23	-3.94	-0.52	
Language				0.381				0.007
Official Swiss language (ref)	0				0			
Other language	0.20	-0.25	0.64		2.22	0.63	3.80	
Continent of parental origin^‡^				0.025				0.018
Western Europe (ref)	0				0			
Other	0.43	0.05	0.80		1.62	0.28	2.97	
Age at T1DM manifestation (years)				<0.001				0.092
<5 (ref)	0				0			
5 to <10	-0.27	-0.63	0.10		1.09	-0.26	2.44	
10 to 16	-0.93	-1.38	-0.49		1.80	0.11	3.49	
Duration of T1DM (years)				<0.001				0.844
<2 (ref)	0				0			
2 to <5	0.98	0.60	1.35		-0.79	-2.66	1.07	
5 to <10	1.21	0.81	1.60		-0.84	-2.80	1.11	
>=10	1.48	0.93	2.03		-0.69	-3.16	1.79	
Modality of insulin therapy				0.268				<0.001
MDI (ref)	0				0			
CSII	0.07	-0.29	0.43		2.62	1.45	3.78	
CT	0.47	-0.10	1.03		-0.58	-2.69	1.52	
Continuous glucose monitoring				0.141				0.153
No (ref)	0				0			
Yes	-0.24	-0.55	0.08		0.82	-0.31	1.96	

Abbreviations: CI: confidence interval; Coef: coefficient; CSII: continuous subcutaneous insulin infusion; CT: conventional therapy; MDI: multiple daily injections; ref: reference category; T1DM: type 1 diabetes mellitus. ^†^Regressions with number of healthcare contacts as response variable include only patients with a T1DM duration of >1 year (*N* = 152). ^‡^Parents were from the following regions: Europe (Western, *n* = 140, *n* = 131 of those from Switzerland; Northern, *n* = 1; Southern, *n* = 16; Eastern, *n* = 2), Asia (Western, *n* = 6; Southern, *n* = 4; Eastern, *n* = 1), Africa (Western, *n* = 1; Northern, *n* = 2; Eastern, *n* = 2), and North America (Northern, *n* = 2; Carribean, *n* = 1).

**Table 3:** Comparison of study characteristics between patients surveyed in 2008 and 2017/2018.

	Year of survey	
	2008	2017/2018
Size of study population (*N*)	152	178
Age at T1DM manifestation (years)		
Median (P25; P75)	6.9 (3.2; 10.6)	6.7 (4.2; 9.7)
Duration T1DM (years)		
Median (P25; P75)	3.4 (1.3; 6.5)	4.0 (1.8; 7.1)
BMI (SDS)		
Median (P25; P75)	0.2 (-0.4; 0.7)	0.3 (-0.3; 0.8)
Modality of insulin therapy		
CSII, *n* (%)	18 (11.8)	47 (26.4)
CT, *n* (%)	66 (43.4)	19 (10.7)
MDI, *n* (%)	68 (44.7)	112 (62.9)
HbA1c (%)		
Mean (SD)^a^	7.8 (1.4)	7.9 (1.0)
<7.5, *n* (%)	40 (26.3)	59 (33.1)
Full time equivalent		
Physicians	3.5	3.5
Nurses	0.6	1.8

Abbreviations: CSII: continuous subcutaneous insulin infusion; CT: conventional therapy; MDI: multiple daily injections; SD: standard deviation; SDS: SD score; T1DM: type 1 diabetes mellitus. ^a^*P* = 0.451 derived from two sample *t*-test comparing mean HbA1c (%) values between 2008 and 2017/2018.

## Data Availability

The data that support the findings of this study are available from the corresponding author upon reasonable request.
